# The naive brain detects face-to-face biological motion

**DOI:** 10.1016/j.isci.2026.116695

**Published:** 2026-07-10

**Authors:** Anastasia Morandi-Raikova, Mirko Zanon, Giorgio Vallortigara

**Affiliations:** 1 Center for Mind/Brain Sciences, University of Trento, Rovereto, Italy

**Keywords:** Social predisposition, animacy detection, agency perception, pallial processing, evolution of social cognition

## Abstract

Detecting agents and their interactions is a cornerstone of social cognition. Vertebrates are known to possess innate sensitivity to animacy cues, such as self-propelled or biological motion, supported by subpallial circuits within the social behavior network (SBN). However, the neural mechanisms distinguishing the detection of animate motion from the recognition of socially meaningful relations remain unclear. Using visually naive female domestic chicks, we investigated the brain regions activated when observing point-light displays of hens moving face-to-face or back-to-back, a behavioral paradigm previously shown to reveal spontaneous sensitivity to social interaction. We found selective immediate early-gene expression in the nidopallium caudolaterale, homologous to the mammalian prefrontal cortex, but not in the nucleus taeniae of the amygdala and septum, subpallial areas in the SBN. This pattern suggests that while basic sensitivity to animate motion may rely on evolutionarily ancient subpallial circuits, the detection of socially relevant relations engages higher order pallial processing.

## Highlights

• Face-to-face biological motion recruits higher order pallial regions in chicks
• Social behavior network regions are not involved
• Social information processing shows right-hemisphere specialization

## Graphical abstract

**Figure:**
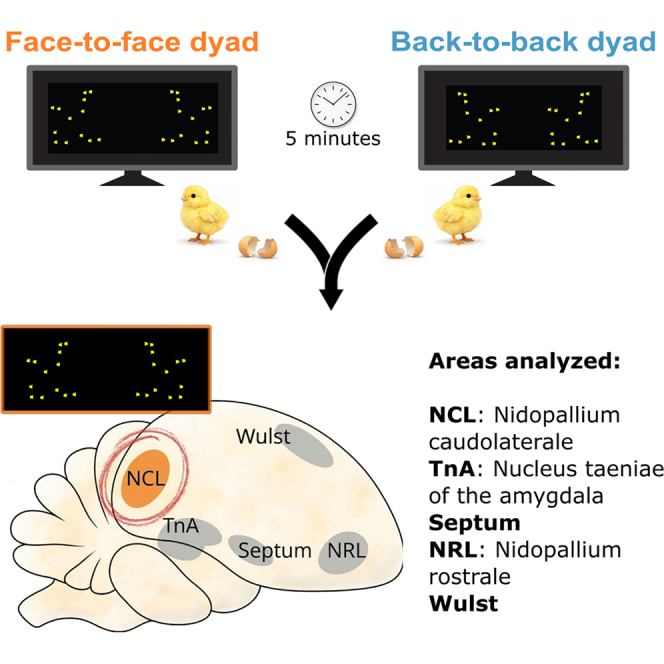

## Teaser

Neuroscience; Behavioral neuroscience; Developmental biology

## Introduction

Sensitivity to face-to-face stimulus configurations, arrangements that likely signal social interaction, emerges early in human development^1^ and has also been demonstrated in macaque monkeys^2^ and newly hatched chicks.^3^ This early predisposition suggests that the detection of potential social engagement may represent a fundamental component of vertebrate social cognition. The ability to perceive two agents facing one another, rather than oriented back-to-back, may thus serve as a primitive cue for recognizing potential interaction.

Beyond static configurations, sensitivity to signals of animacy can arise solely from motion cues, even in the absence of prior information. Movements that are self-propelled or display changes in speed and direction are spontaneously perceived as animate.^4^^,^^5^ This capacity appears to be widespread among vertebrates and present from the very beginning of life.^6^^,^^7^^,^^8^ Research on biological motion perception has shown that organisms can extract rich information about agency and intentionality from sparse motion cues alone.^9^^,^^10^^,^^11^^,^^12^^,^^13^ Across many species, from humans to non-human primates, birds, and fish, point-light displays have been used to demonstrate that the coordinated kinematics of motion are sufficient to evoke the perception of a living agent.^14^^,^^15^^,^^16^^,^^17^^,^^18^^,^^19^ The spontaneous attraction of visually naive animals to such stimuli (despite the absence of shape, color, or texture information) reveals that sensitivity to biological motion is an evolutionarily ancient and likely innate feature of vertebrate brains.

Importantly, *animacy*, *agency*, and *social interaction* are related but distinct aspects of social perception. Animacy refers to the perception of something alive or self-propelled, often inferred from motion cues. Agency involves attributing goal-directed behavior to an entity, whereas social interaction requires recognition of relations between two or more agents. Although detecting animacy perception from motion can contribute to the attribution of agency,^20^ the detection of social interaction relies on perceiving structured relationships between multiple agents. Thus, stimuli depicting two individuals facing one another may specifically provide cues to potential social engagement, beyond the basic perception of biological motion.

At the neural level, the detection of animacy has been linked to subpallial circuits belonging to the so-called social behavior network (SBN).^21^^,^^22^^,^^23^ In chicks, exposure to simple motion cues such as abrupt speed changes, a key signature of self-propelled movement, elicits activation in the septum, a major component of this network.^23^ This supports the view that sensitivity to animacy can arise from low-level kinematic information. In addition, responses to biological motion have been associated with activation in the preoptic area of the hypothalamus and in the nucleus taeniae of the amygdala (TnA).^24^^,^^25^ These subpallial structures, which are evolutionarily conserved across vertebrates, appear to encode the presence of animate agents through motion-based cues, forming the neural substrate for innate animacy detection.

However, when motion cues convey not only animacy but also relational information between agents, as in the case of two individuals facing one another, the percept may shift from mere detection of animate motion to the recognition of a potential social interaction. In such cases, animals respond not simply to the presence of animate entities but to the *relations* between them. This is well known in humans when comparing bodies positioned face-to-face vs. back-to-back^1^^,^^26^: for example, infants show a significant difference in looking times toward the two types of dyads,^1^ which parallels the adult preference for face-to-face configurations.^2^ The same preference is present in monkeys.^2^ More recently, research in visually naive chicks showed that these animals preferentially approach face-to-face, rather than back-to-back, point-light displays of conspecifics, despite both configurations containing equivalent cues of biological motion.^3^ This suggests that animals are sensitive to higher order social contingencies beyond simple animacy and independently of the particular visual cues that generate them (see Goupil et al., Papeo, and Papeo et al.^1^^,^^26^^,^^27^ for specific investigations on this topic).

Point-light biological motion stimuli, particularly in face-to-face vs. back-to-back configurations, provide a well-controlled framework to investigate the neural correlates of biological motion and socially meaningful relations between agents. This design allows disentangling their interplay in a systematic manner by comparing responses to the two types of dyads. Based on previous evidence, we hypothesize that subpallial regions involved in animacy detection may respond similarly to both configurations, whereas differences related to the perception of social interaction between agents may emerge within pallial structures, particularly in regions functionally analogous to the mammalian prefrontal cortex (PFC). Using newly hatched, naive chicks as an animal model further allows testing whether these neural correlates are innate.

## Results

### Face-to-face point-light dyads selectively increase activity in the right NCL

**Figure 1:**
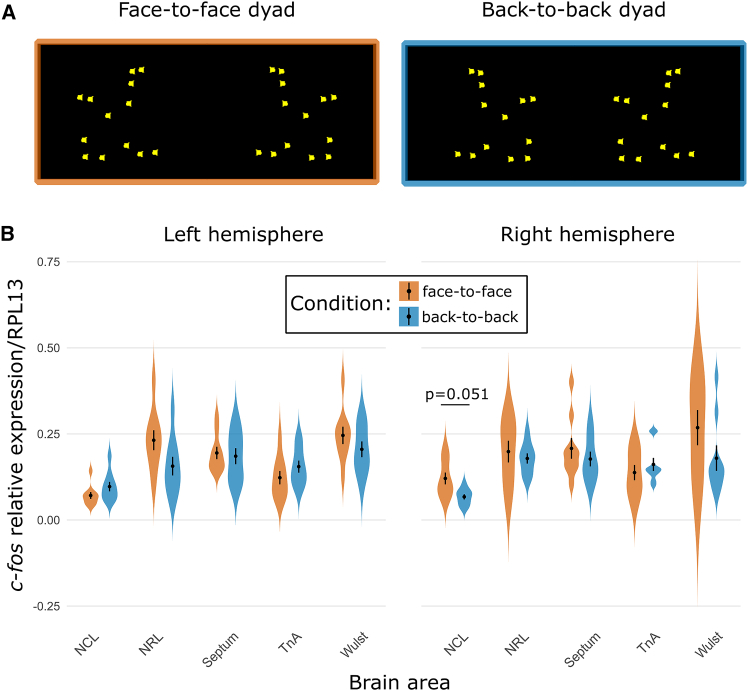
Stimuli and *c-fos* activity (A) Static schematic of the stimuli used in the experiment, with the face-to-face (left) and back-to-back (right) dyads, which in the real experiment were displayed in dynamic configurations so that the silhouette points were moving according to biological motion. (B) Results of the experiment, with violin plots for each hemisphere and brain area representing *c-fos* activity distribution across subjects (9 + 9 chicks) for the different experimental conditions (color coded). In black, the chicks’ average *c-fos* activity with SEM bars. Permutation ANOVA post hoc *p* value is reported.

The expression levels of c-fos were quantified via qPCR (see STAR Methods and Table 1). A series of permutation-based mixed ANOVAs (10,000 iterations), with condition (face-to-face vs. back-to-back point-like dyads) and hemisphere (left vs. right) as factors, were conducted separately for each of the five brain regions of interest (NCL [nidopallium caudolaterale], NRL [nidopallium rostrolaterale], TnA, septum, and visual Wulst). Hemisphere was included as a factor because previous work has shown strong hemispheric specialization in the processing of socially relevant stimuli in chicks and other birds.^28^^,^^29^^,^^30^ No significant effects were found in the NRL, TnA, septum, or Wulst. In contrast, the analysis of the NCL revealed a significant condition × hemisphere interaction (F_1,16_ = 9.68, permutation *p* = 0.0050, η_p_^2^ = 0.38 [95% confidence interval (CI): 0.08, 1.00]; Figure 1B).

**Table 1:** Primer sequences used for RT-qPCR

Gene primer	Sequence
*c-fos* Fw	GTGTTCCTGGCAATATCGTG
*c-fos* Rw	TCAGACCACCTCAACAATGC
RPL13 Fw	TCGTGCTGGCAGAGGATTC
RPL13 Rw	TCGTCCGAGCAAACCTTTTG

Post hoc Wilcoxon rank-sum tests, adjusted using the Benjamini-Hochberg (false discovery rate [FDR]) correction, revealed that *c-fos* activity showed a strong trend toward higher activation in the face-to-face compared to the back-to-back condition within the right NCL hemisphere (W = 68, p_FDR_ = 0.051). This difference represented a substantial increase in neural activation (mean differences [MD] = 0.053) and a strong effect size (r_RB_ = 0.68, 95% CI: [0.27, 0.88]). Moreover, in the face-to-face condition, the activity was higher in the right than in the left NCL hemisphere (W = 12, p_FDR_ = 0.051, MD = 0.049). This hemispheric asymmetry was characterized by a robust effect size (r_RB_ = 0.70, 95% CI: [0.32, 0.89]), confirming that social orientation selectively recruits the right associative forebrain.

### Stronger right NCL activation is associated with greater left visual field use

Before investigating brain-behavior relationships, we analyzed the distribution of the field-of-view (FOV) lateralization scores. The overall behavioral lateralization index did not significantly differ between the face-to-face and back-to-back groups (Wilcoxon rank-sum test W = 49, *r*_RB_ = 0.16 [95% CI: −0.37, 0.61], *p* = 0.596), indicating that the experimental conditions did not drive a global shift in baseline visual field use.

**Figure 2:**
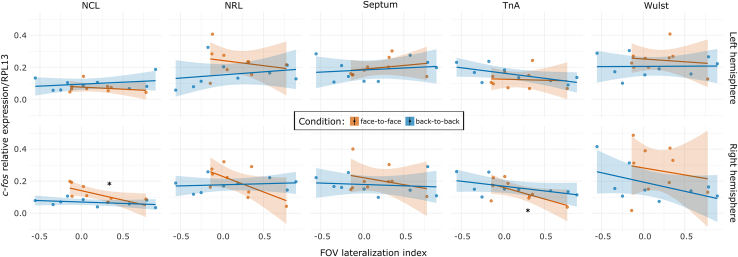
Correlation between brain activity and behavioral output Correlation lines for the *c-fos* activity and the field-of-view (FOV) lateralization score, for each brain area and hemisphere (experimental conditions are color coded). A negative lateralization score indicates the use of the left FOV (and thus the left eye), while a positive one indicates the use of the right FOV. ∗ indicates a *p* ≤ 0.05 for the Pearson correlations.

To assess whether neural activation was related to behavioral outputs, we correlated *c-fos* activity with the FOV lateralization score, which quantifies the preferential visual field use (negative values indicating left-eye bias and positive values right-eye bias, Figure 2). To account for the sample sizes, we utilized Pearson correlations (r) and computed bootstrapped 95% confidence intervals (10,000 iterations).

A significant negative correlation was found in the right NCL hemisphere in the face-to-face condition (r = −0.69, 95% CI: [–0.97, −0.46], *p* = 0.038), indicating that stronger activation was associated with greater left FOV use. Additional correlations that did not reach statistical significance, and therefore should be interpreted with more caution, were observed in the right NRL hemisphere of the face-to-face condition (r = −0.63, 95% CI [–0.95, 0.33], *p* = 0.067). An anticorrelation was detected in the right TnA hemisphere of the face-to-face condition (r = −0.67, 95% CI: [–0.97, 0.02], *p* = 0.049), while a similar correlation that did not reach statistical significance was observed in the left TnA hemisphere of the back-to-back condition (r = −0.64, 95% CI: [–0.94, −0.12], *p* = 0.062).

We stress that these analyses should be considered exploratory, especially given the small final sample and the presence of borderline/non-significant associations.

## Discussion

Our findings reveal differential activation of the NCL in visually naive, newly hatched, female chicks following their first exposure to social interactions. Specifically, the right NCL showed a strong trend toward higher activation when female chicks viewed biologically moving, interacting dyads (face-to-face condition) compared with non-interacting dyads (back-to-back condition). A similar pattern of lateralized responsiveness was observed in the NRL, although this effect did not reach statistical significance, suggesting a comparable lateralized responsiveness in this associative area.

No significant differences between stimulus conditions were detected in the SBN regions examined here (septum and TnA), nor in primary visual regions such as the visual Wulst.

Although no group-level difference in visual field use was observed, activation in the right NCL was stronger when female chicks viewed the interacting stimulus with their left eye, indicating a possible right-hemispheric specialization for processing socially contingent motion. A similar but weaker pattern emerged in the right NRL. Interestingly, within the right TnA, chicks primarily using their left eye exhibited greater *c-fos* activation in the face-to-face condition, while a similar non-significant pattern of activation was present for the back-to-back dyad in the left hemisphere.

Functionally, the NCL is considered analogous to the mammalian PFC and is involved in executive functions such as decision-making, attention control, and behavioral flexibility, capacities essential for interpreting socially relevant cues.^31^ In corvids, NCL activity has been linked to cognitively controlled, socially meaningful vocalizations,^32^ supporting its role in integrating executive and social processing. Evidence from one-week-old, face-naive domestic chicks further indicates that NCL neurons selectively respond to face-like configurations but not to rearranged or isolated features,^33^ suggesting that this region contributes early to the detection of socially contingent stimuli.

Accordingly, the increased activation observed in the NCL during exposure to the face-to-face configuration likely reflects the higher social relevance of stimuli conveying relational cues between agents. Our findings may therefore reflect an early sensitivity to cues of social interaction rather than to agency alone. In the present paradigm, both conditions contained equivalent biological motion cues and thus conveyed information about animacy. However, the face-to-face configuration additionally provides relational cues that may arise from motion cues produced by single agents, while social interaction perception depends on the structured relations between multiple agents. These processes may rely on partially distinct neural mechanisms, consistent with findings suggesting that animacy perception from motion involves multiple interacting dimensions.^20^^,^^34^ Consistent with this framework, early sensitivity to biological motion cues has been linked to subpallial circuits of the SBN, whereas the detection of socially meaningful relations between agents may recruit higher order pallial regions such as the NCL. The right-lateralized activation observed aligns with the established right-hemispheric dominance in processing social information.^28^^,^^29^^,^^30^ In chicks, the left-eye-system/right-hemisphere is involved in social learning, hierarchy formation, predator detection, and fear responses.^29^^,^^35^^,^^36^ Moreover, the degree of cerebral asymmetry has been linked to social integration within groups, where stronger lateralization correlates with greater social connectedness.^37^ Collectively, these findings support the idea that lateralization enhances social competence and adaptive efficiency.

Although NRL activation did not reach significance, a tendency toward greater responsiveness was observed, consistent with evidence implicating the rostral nidopallium in social information processing. In crows, the rostral nidopallium shows increased activation in response to familiar human faces,^38^ suggesting its involvement in the recognition of socially relevant individuals. Anatomically, this region is strongly interconnected with associative visual areas such as the entopallium and mesopallium^39^ and receives input from both the tectofugal and thalamofugal visual pathways,^40^ supporting its role as an integrative hub for socially meaningful visual information. Although most studies have focused on the NCL, our results highlight the need to consider the NRL in future analyses of avian social cognition.

Interestingly, no modulation of the visual Wulst was detected between stimulus conditions or hemispheres. Given that the Wulst primarily supports visuomotor and spatial processing,^41^^,^^42^ this lack of differential activation is expected. Both experimental stimuli were matched for visual motion, luminosity, and dot configuration, differing only in their social meaning, factors that would probably differentially engage associative rather than primary visual regions.

Interestingly, previous studies showed that the face-to-face positioning of bodies impacts the very early, preattentive stages of visual perception^26^^,^^43^ up to visual memory,^44^^,^^45^ with neuroimaging studies in humans showing how body-selective regions of the visual cortex respond more strongly to multiple interacting bodies than to unrelated bodies.^46^^,^^47^

No significant effects were detected in either the septum or TnA in our experiment. These nuclei, together with the preoptic area, hypothalamic regions, and parts of the midbrain, form the SBN, a conserved vertebrate circuit regulating diverse social behaviors.^22^^,^^23^^,^^24^^,^^48^ Previous studies have shown septal activation during first exposure to live conspecifics or to self-propelled animate stimuli.^23^^,^^24^ The absence of septal activation in the present study is consistent with recent evidence indicating that the septum does not respond to point-light biological motion,^25^ as no difference in activation was found when chicks viewed point-light displays of biologically moving versus rotating hens.

The TnA, typically implicated in processing social reward and fear,^49^^,^^50^^,^^51^ also did not differentiate between the two stimulus types. However, the eye-dependent lateralized pattern observed did not reach statistical significance and should therefore be interpreted cautiously, although it is consistent with the possibility that TnA responds more generally to biological motion rather than to its social contingency.^25^ This aligns with prior findings showing differential *c-fos* expression when chicks viewed biological vs. non-biological motion,^24^ where direction of gene regulation may have reflected distinct cellular responses (e.g., excitatory vs. inhibitory neurons) rather than behavioral preference per se.

Overall, these results indicate that within the regions examined here, the septum and TnA did not show detectable differences in activation between the two conditions. Because the present study examined only a subset of nuclei belonging to the SBN, these results should not be interpreted as evidence that the SBN as a whole is not involved in processing socially meaningful motion cues. Instead, they suggest that the relational information distinguishing the two stimuli preferentially engages higher order pallial regions such as the NCL.

Our findings indicate preferential engagement of the NCL in visually naive female chicks when processing stimuli conveying socially meaningful relations between agents. This finding highlights the involvement of higher order associative pallial regions in interpreting socially contingent motion, even in the complete absence of prior visual experience. In contrast, components of the SBN and primary visual areas appear to respond to more fundamental aspects of perception—such as the detection of biological motion cues associated with animacy—rather than to the social meaning embedded within those movements. Previous studies in naive chicks have shown that subpallial regions of the SBN respond to motion cues characteristic of animate stimuli, including self-propelled movement and biological motion.^23^^,^^24^^,^^25^

These results suggest that pallial associative regions such as the NCL may contribute to the processing of socially contingent motion cues early in development, even in the absence of prior visual experience. Such mechanisms may provide a neural foundation for the rapid emergence of social competencies in precocial species.

More broadly, the lateralized engagement of the NCL observed here supports the view that hemispheric specialization in social processing is an evolutionarily conserved strategy that enhances the efficiency and accuracy of social perception across vertebrate taxa.

### Limitations of the study

A limitation of the present study is that our analysis focused on a restricted set of brain regions, primarily within higher order associative pallial areas and selected nuclei of the SBN. Future work should extend the investigation to additional integrative regions that may contribute to the processing of socially contingent motion.

Although our findings revealed robust patterns of lateralized activation in the NCL, other regions such as the NRL and TnA exhibited moderate effect sizes that did not reach statistical significance. Future replication with larger cohorts will be essential to determine whether these non-significant patterns represent subtle functional specializations or stochastic biological noise, particularly during the early stages of social neural processing.

Another limitation of the present study is the absence of additional visual control conditions, such as inverted point-light displays. Although the two experimental stimuli were matched for motion kinematics and differed only in the relational orientation between agents, future studies including such controls could further disentangle the contribution of low-level motion processing from higher level sensitivity to socially meaningful configurations.

A further limitation is that both stimuli contained biological motion cues and therefore did not directly contrast animate with non-animate motion. Although previous studies have shown that subpallial regions of the SBN respond to motion cues characteristic of animate stimuli, including self-propelled movement and biological motion,^23^^,^^24^^,^^25^ future studies including additional control conditions could further clarify the respective contributions of biological motion processing and social interaction cues.

Finally, the present study relied exclusively on female chicks. Although this choice was motivated by previous behavioral findings showing sex-specific effects with these stimuli,^3^ future studies should include both sexes to determine whether similar neural mechanisms underlie the processing of socially contingent motion in male chicks and to what extent sex modulates lateralized responses.

## Resource availability

### Lead contact

Further information and requests should be directed to and will be fulfilled by the lead contact, Anastasia Morandi-Raikova (amorandi.raikova@unitn.it).

### Materials availability

This study did not generate new unique reagents or new animal lines.

### Data and code availability

• Data are available in Figshare: https://doi.org/10.6084/m9.figshare.30490487.
• Code used for the analysis of this work is available in Figshare: https://doi.org/10.6084/m9.figshare.30490487.
• Any additional information required to reanalyze the data reported in this paper is available from the corresponding contact upon request.

## Acknowledgments

This project was funded by PRIN – Progetti di rilevante Interesse Nazionale 2022-PNRR grant agreement 2022WX3FM5: “Searching for sensitivity to stimulus statistics: Evolutionary mechanisms, development and critical periods underlying efficient neural coding” to G.V.

## Author contributions

Conceptualization, A.M.-R., M.Z., and G.V.; setup implementation, A.M.-R. and M.Z.; methodology, A.M.-R., M.Z., and G.V.; investigation, A.M.-R., M.Z., and G.V.; visualization, A.M.-R. and M.Z.; funding acquisition, G.V.; supervision, G.V.; writing – original draft, A.M.-R. and M.Z. All authors reviewed and edited the final version of the manuscript.

## Declaration of interests

The authors declare no competing interests.

## Declaration of generative AI and AI-assisted technologies in the writing process

During the preparation of this work, the authors used ChatGPT only to improve the English text and readability of some sentences. After using this tool, the authors reviewed and edited the content as needed and take full responsibility for the content of the publication.

## STAR★Methods

### Key resources table

**Table:** 

REAGENT or RESOURCE	SOURCE	IDENTIFIER
**Critical commercial assays**		

Arcturus PicoPure RNA Isolation Kit	ThermoFisher Scientific	Cat# KIT0204
SuperScript™ VILO™ cDNA Synthesis Kit	Invitrogen, ThermoFisher Scientific	Cat#11754050
PowerUp™ SYBR™ Green Master Mix (2×)	Applied Biosystems, ThermoFisher Scientific	Cat#A25742

**Experimental models: Organisms/strains**		

Domestic chick (*Gallus gallus domesticus*, Ross 308 strain, female)	Local commercial hatchery (Passirano, Brescia, Italy)	Ross 308 strain

**Oligonucleotides**		

c-fos qPCR Forward primer: GTGTTCCTGGCAATATCGTG	Sigma-Aldrich	Oligo#8823686178-10/0
c-fos qPCR Reverse primer: TCAGACCACCTCAACAATGC	Sigma-Aldrich	Oligo#8823686178-20/0
RPL13 qPCR Forward primer: TCGTGCTGGCAGAGGATTC	Sigma-Aldrich	Oligo#8823686178-30/0
RPL13 qPCR Reverse primer: TCGTCCGAGCAAACCTTTTG	Sigma-Aldrich	Oligo#8823686178-40/0

**Deposited data**		

Data for cfos expression and FOV used	This paper	https://doi.org/10.6084/m9.figshare.30490487
R script for statistical data analysis	This paper	https://doi.org/10.6084/m9.figshare.30490487

**Software and algorithms**		

MATLAB version R2023b	MathWorks	https://it.mathworks.com/products/matlab.html
RStudio version 2023.06.0 + 421	Posit Software, PBC	https://posit.co/download/rstudio-desktop/
DeepLabCut	Mathis et al.^52^	http://www.mackenziemathislab.org/deeplabcut
ImprintSchedule (stimuli presentation tool)	Zanon et al.^53^	https://link.springer.com/article/10.1007/s00422-021-00886-6

**Other**		

Tissue-Tek OCT Compound	Sakura Finetek	Cat#4583
SuperFrost Plus microscope slides	ThermoFisher Scientific	Cat#J1800AMNZ
Leica CM1860 UV cryostat	Leica Biosystems	Model CM1950
NanoDrop™ OneC spectrophotometer	ThermoFisher Scientific	Model OneC
C1000 Touch™ Thermal Cycler	Bio-Rad	Cat#1851197
CFX96™ Real-Time PCR Detection System	Bio-Rad	Cat#1855196

### Experimental model and study participant details

#### Animals

Subjects were 35 laboratory-hatched domestic chicks (*Gallus gallus domesticus*, Ross 308 strain). The limited number of subjects was selected to remain consistent with previous studies using similar methodologies^24^^,^^25^^,^^50^ and to comply with ethical regulations based on the 3Rs principles, minimizing the number of animals used while ensuring scientific validity.

From the total *n* = 35, a subset of 15 chicks (43%) was preliminary discarded because escaping (*n* = 10) or sleeping for most (>2 min) of the time (*n* = 5). We remained with 20 chicks (*n* = 10 per group) for the brain analysis. After outlier exclusion (*c-fos* activity above 2 sigma; *n* = 2), we got *n* = 9 chicks per group.

Only female chicks were tested, as in a previous behavioral study using the same stimuli, a sex-specific effect was reported, specifically, a significant difference in approach rate was observed only in females.^3^ Fertilized eggs were obtained from a local commercial hatchery (Passirano, Brescia, Italy) and incubated in complete darkness within a Marans P140TU-P210TU incubator at a constant temperature of 37.7°C and 60% relative humidity. After hatching, chicks were maintained in total darkness inside the incubators until testing, under the same temperature and humidity conditions. All procedures were conducted in complete darkness to prevent any visual experience before and after testing. All experimental procedures complied with current Italian and European Union legislation on the ethical use of animals in research. The study was approved and licensed by the Italian Ministry of Health, Department of Food, Nutrition and Veterinary Public Health (permit no. 552/2025-PR; response to protocol 07753.52).

### Method details

#### Apparatus and stimuli

The experiment was conducted in a dark room. The apparatus consisted of a rectangular cage (45 × 60 × 60 cm; L × W × H) open on one side to accommodate a video monitor (LCD BenQ XL2410T, 23.6 inches, 1920 × 1080 pixels, 120 Hz) used for visual stimulation. In front of the screen, at a distance of 20 cm, a black plastic panel (1 × 30 cm; W × H) was positioned with a circular opening (2.5 cm in diameter) through which the chick’s head protruded. The opening was centered horizontally and located 6 cm from the bottom edge of the panel. A soft weight was gently placed behind each chick to maintain its position without causing discomfort. This arrangement ensured that chicks viewed the stimuli from a consistent fixed position during the 5-min visual exposure while helping prevent them from moving away from the display.

The only source of illumination in the room was the monitor itself, which displayed the visual stimuli. The stimuli consisted of face-to-face and back-to-back dyads of walking point-light silhouette hens (Figure 1A), displayed through MATLAB Psychtoolbox-3^54^^,^^55^^,^^56^ at 15 frames per second. The two stimulus conditions were constructed using identical point-light biological motion sequences, differing only in the relative spatial orientation of the two agents (face-to-face vs. back-to-back). Consequently, the stimuli were matched for dot number, motion kinematics, luminance, and overall biological motion content, while differing only in the relational configuration between the agents. The whole dimension of a hen dyad was of 20 (length) X 8 (height) cm on screen; from the chick’s position (a distance of 20 cm), the complete stimulus subtended a visual angle of approximately 53.1 ° × 22.6 °; the single point (of side 0.3 cm) subtended 0.9° (corresponding to 0.59 cycles/deg), below chicks’ visual acuity limit (1.17 cycles/deg at 1dpf^57^). The stimuli are the same used in Zanon et al., 2024,^3^ which originate from the legacy point-light animations first generated by Vallortigara et al. (2005).^14^ These animations were originally created by digitizing the joint positions of a real hen walking on a treadmill, resulting in a fluid biological motion display with no overall translational movement across the screen. The point-light joints were rendered as yellow squares to maintain exact methodological continuity with recent baseline behavioral literature. While the native frame rate of these original legacy files is 15 fps, previous research has extensively demonstrated that this temporal resolution is entirely sufficient to trigger robust, spontaneous biological motion perception in newly hatched chicks.^3^^,^^14^^,^^25^^,^^58^

Each chick was exposed to a single dyad (10 chicks face-to-face, 10 chicks back-to-back).

A webcam mounted above the monitor recorded the chick’s behavior from the front. The webcam feed was connected to a screen in the same room, allowing real-time monitoring of each chick’s orientation and attention toward the stimulus. Chicks that managed to escape the position or slept for more than 2 min during the 5-min exposure were excluded from further brain analysis. All videos were recorded for offline behavioral analyses.

#### Behavioral tracking

Head movements during the 5-min experiment were recorded and subsequently tracked offline using DeepLabCut.^52^ From this tracking data, we computed the eye and beak position relative to the central frontal stimulus. We then calculated the percentage of the stimulus area observed by the left versus the right chick’s field of view (FOV) for each time frame. These FOV calculations used the central, straight head position as a baseline Vallortigara et al.^59^

#### Tissue preparation

Fifteen minutes after visual stimulation, each chick was euthanized with an intramuscular injection of 0.05 mL ketamine/xylazine solution (1,1 Ketamine 10 mg/mL + xylazine 2 mg/mL) per 10 g of body weight. 5 min after the injection, the animal was confirmed unresponsive, and the head was immediately severed and placed on ice. The skull was then secured in a stereotaxic head holder and surrounded by ice to minimize RNA degradation. Brains were extracted following the anatomical procedures described in the chicken brain atlas (Kuenzel & Masson, 1988), ensuring that all sections maintained the same orientation as depicted in the atlas. The two brain hemispheres were processed separately and immediately placed on dry ice before being stored at −80°C shortly after extraction. One day prior to tissue sectioning, brains were transferred to −20°C. Three series of 60 μm coronal sections were cut from regions of interest using a cryostat (Leica CM1850 UV). All sections were collected directly onto microscope slides (Super-Frost Plus; ThermoFisher Scientific) and stored at −20°C until further processing. Sections from the first series were used for RNA extraction, while the remaining two series were kept as backups.

The regions of interest—Wulst (A13.6–A10.2), TnA (A7.6–A6.4), Septum (A9.2–A8.4), NCL (A10.0–A7.2), and NRL (A12.4–A11.2)—were microdissected (Li et al., 2018) using sterile 10-μL pipette tips. Total ribonucleic acid (RNA) was extracted using the Arcturus PicoPure RNA Isolation Kit (Thermo Fisher Scientific) following the manufacturer’s protocol. RNA purity (A260/A280 and A260/A230 ratios) and concentration were assessed with a NanoDrop spectrophotometer (NanoDrop OneC; Thermo Fisher Scientific). Complementary DNA (cDNA) was synthesized using the SuperScript VILO cDNA Synthesis Kit (Invitrogen, Thermo Fisher Scientific) according to the manufacturer’s instructions.

#### qPCR

qPCR was performed to measure the expression levels of c-fos and RPL13 using gene-specific forward and reverse primers (Table 1). Reactions were carried out in duplicate using the PowerUp SYBR Green Master Mix (2×) on a CFX96 Real-Time PCR Detection System (Bio-Rad). Gene expression levels were quantified using the ΔCq method (see Messina et al., 2020). Expression data were normalized to the RPL13 reference gene, and relative expression values were calculated accordingly.

### Quantification and statistical analysis

All statistical analyses were performed in R (version 2023.06). Data were obtained from five brain regions of interest (Nidopallium caudolaterale, NCL; Nidopallium rostrale, NRL; nucleus taeniae, TnA; Septum; and Wulst), each sampled in both hemispheres (left, right) and under two experimental conditions (face-to-face dyad and back-to-back dyad; between subjects). To ensure robust estimation and avoid strong noise influences a strict outlier exclusion protocol was implemented (see e.g.,^60^^,^^61^). Because chicks are highly sensitive to their surroundings, transient environmental distractions during the procedure can cause off-target *c-Fos* activation that obscures the target stimulus-driven response. To filter out this possible biological noise, alongside potential localized histological artifacts, any subject exhibiting *c-Fos* activity deviating more than 2 standard deviations from their respective group-by-area-by-hemisphere mean was removed entirely from the dataset. This uniform exclusion resulted in a final sample of 9 chicks per experimental group (from an initial cohort of 10 per condition).

Prior to statistical testing, data distributions were examined for normality using the Shapiro–Wilk test and for homogeneity of variance using Levene’s test. Since assumptions were not fully met across several regions, non-parametric permutation analyses were adopted.

For each brain region, we performed a mixed-design permutation ANOVA (10,000 iterations) using the *aovperm* function from the *permuco* R package. The models included *Condition* (face-to-face and back-to-back dyads) and *Hemisphere* (left, right) as fixed factors, with individual animals as a random factor to account for within-subject dependence. Unlike standard parametric ANOVAs, this resampling approach builds an empirical null distribution by repeatedly shuffling group labels across the observed data, providing stable *p*-values without assuming normality. To quantify the magnitude of the variance explained by our factors, we calculated partial eta-squared (η_p_^2^). This metric represents the proportion of total variance attributable to a specific effect (e.g., the Condition × Hemisphere interaction) after excluding variance from other factors, calculated as:

η_p_^2^ = SS_effect_ /(SS_effect_ +SS_error_)

where SS_effect_ is the sum of squares for the effect of interest and SS_error_ is the residual sum of squares.

When significant effects emerged, pairwise post-hoc comparisons were conducted using Wilcoxon rank-sum tests. To characterize the strength of these differences, we reported raw Mean Differences (MD) and the rank-biserial correlation (r_RB_) with 95% confidence intervals. The r_RB_ is a non-parametric effect size that standardizes the difference in the proportion of favorable evidence between two groups on a scale from −1 to 1:

r_RB_ = (U_1_ -U_2_)/(U_1_ +U_2_)

where U represents the Mann-Whitney U statistic.

To control the false discovery rate (FDR) associated with multiple comparisons, *p*-values were adjusted using the Benjamini–Hochberg correction. This procedure was preferred over more conservative methods (e.g., Holm-Bonferroni) to maintain an appropriate balance between Type I and Type II error rates in our biological sample.

To investigate the relationship between neural activity and visual behavior, we computed correlations between *c-Fos* activation and a lateralization index derived from behavioral tracking. Head and eye positions were tracked using DeepLabCut.^52^ Based on the chick’s head orientation relative to the stimulus, we quantified the proportion of time each chick used the left visual field (L) and right visual field (R) during the 5-min stimulus exposure. A field-of-view (FOV) lateralization score was then calculated for each chick as (R–L)/(R + L). This score reflects the direction and magnitude of visual field bias, with positive values (a score >0) indicating a right-eye bias and negative values (a score <0) a left-eye bias.

Baseline differences in this behavioral index between groups were assessed using a Wilcoxon rank-sum test. Finally, for each brain region, hemisphere, and condition, Pearson correlations (*r*) were computed between individual FOV lateralization scores and mean *c-Fos* activity. To account for the sample size and ensure the reliability of these estimates, 95% confidence intervals for the Pearson coefficients were generated via a bootstrapping procedure with 10,000 iterations using the *boot* package.
